# Pharmacological regeneration of sensory hair cells restores afferent innervation and vestibular function

**DOI:** 10.1172/JCI181201

**Published:** 2024-09-24

**Authors:** Hanae Lahlou, Hong Zhu, Wu Zhou, Albert S.B. Edge

**Affiliations:** 1 Department of Otolaryngology, Harvard Medical School, Boston, Massachusetts, USA.; 2 Eaton-Peabody Laboratory, Massachusetts Eye and Ear, Boston, Massachusetts, USA.; 3 Department of Otolaryngology-Head and Neck Surgery, University of Mississippi Medical Center, Jackson, Mississippi, USA.; 4 Harvard Stem Cell Institute; Cambridge, Massachusetts, USA.

**Keywords:** Otology, Therapeutics, Innervation

## Abstract

The sensory cells that transduce the signals for hearing and balance are highly specialized mechanoreceptors called hair cells that together with supporting cells comprise the sensory epithelia of the inner ear. Loss of hair cells from toxin exposure and age can cause balance disorders and is essentially irreversible due to the inability of mammalian vestibular organs to regenerate physiologically active hair cells. Here, we show substantial regeneration of hair cells in a mouse model of vestibular damage by treatment with a combination of glycogen synthase kinase 3β and histone deacetylase inhibitors. The drugs stimulated supporting cell proliferation and differentiation into hair cells. The new hair cells were reinnervated by vestibular afferent neurons, rescuing otolith function by restoring head translation–evoked otolith afferent responses and vestibuloocular reflexes. Drugs that regenerate hair cells thus represent a potential therapeutic approach to the treatment of balance disorders.

## Abstract 2

Regeneration of hair cells in the inner ear by a drug treatment restored the sense of balance in an experimental model of balance dysfunction.

## Graphical abstract

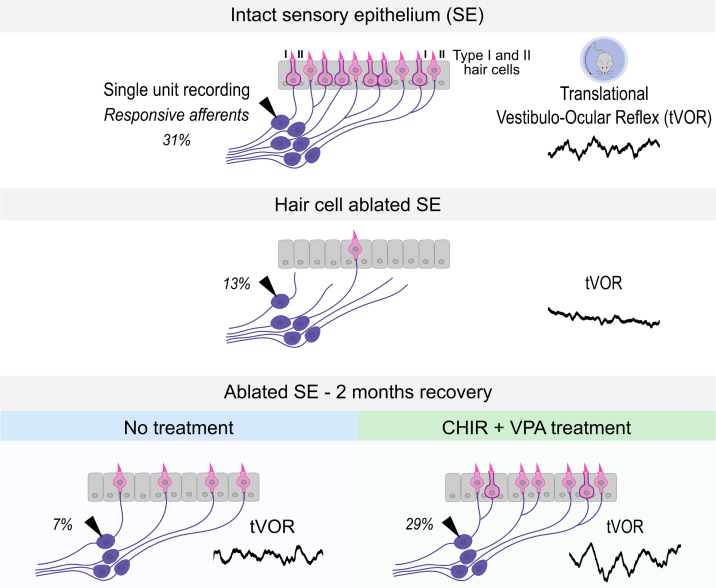

## Introduction

Balance disorders occur in 35% of adults over 40 years of age and are the number one health complaint of individuals over 70 years of age (1, 2). The sense of balance relies on sensory cells called hair cells (HCs), mechanoreceptors in the semicircular canals and otolith organs of the inner ear that sense angular and linear acceleration, respectively. The HCs are interdigitated by supporting cells (SCs) in a sensory epithelium (3), where they are innervated by afferent neurons (4–7). Vestibular sensory cells comprise type I and type II HCs that drive regular and irregular afferent neurons. The neurons are distinguished by their firing patterns and encode the head acceleration signals for transmission to the CNS. HCs are vulnerable to damage from insults such as aging, mutations, acoustic overstimulation, and ototoxic agents, resulting in profound balance deficits (1, 4, 8, 9). SCs are endowed with cellular plasticity that allows some spontaneous regeneration of HCs in response to damage (10–16), but regeneration declines in adulthood in mammals, starkly contrasting with nonmammalian vertebrates, where a continuous cell turnover leads to HC regeneration throughout life (10–23). Type I and type II HCs may emanate from a common progenitor but are thought to result from independent developmental trajectories (24). For both HC types, SCs appear to act as progenitors to regenerate HCs (14, 23, 25). Newly regenerated HCs are exclusively of the type II phenotype in mature utricle (11, 26), but because both HC types are important for detecting head motion, the limited extent of regeneration constitutes an important block to functional recovery (20, 23, 27–29).

Cellular reprogramming using prodifferentiation factors has allowed the application of regenerative approaches to cell replacement by altering the fate of endogenous cells (30), but reprogrammed cells have not been shown to acquire full physiological activity (31). Studies on hearing and balance in the inner ear have relied on the overexpression of the bHLH transcription factor, Atoh1, to reprogram SCs to HCs and have seen partial success in achieving a HC fate but mixed results in restoring mechanosensory function (32). Here, by delivering small molecules into the ear in a mouse model of vestibular HC loss, we found a substantial regeneration of HCs, accompanied by afferent reinnervation and restoration of vestibular function as assessed by single vestibular afferent activities and the vestibuloocular reflex (VOR).

## Results

### Drug-induced HC regeneration in Pou4f3^DTR^-ablated utricles.

**Figure 1:**
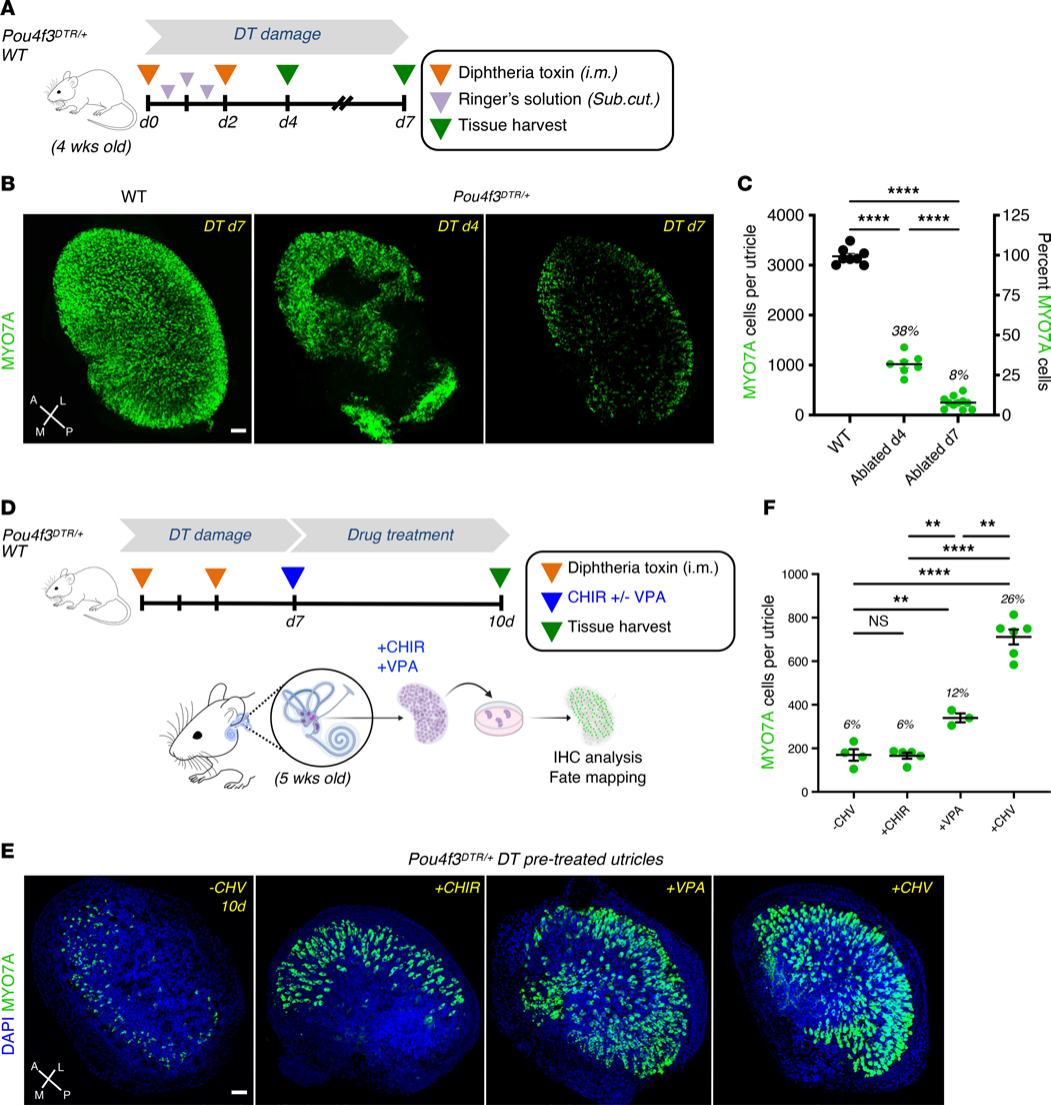
Ex vivo drug treatment enhances HC regeneration in DT-ablated *Pou4f3^DTR/+^* mouse utricle. (**A**) Schematic of DT in vivo damage. Four-week-old WT and *Pou4f3^DTR/+^* mice received 2 intramuscular injections of diphtheria toxin (DT). Utricles were analyzed 4 and 7 days after damage. (**B**) MYO7A immunolabeling of undamaged WT and *Pou4f3^DTR/+^* ablated utricles. (**C**) MYO7A^+^ cell counts of WT and DT-ablated utricles. (**D**) Schematic of in vitro drug treatment. At 7 days after damage, utricles were harvested from WT and *Pou4f3^DTR/+^* mice and cultured in medium without drug (–CHV) or supplemented with CHIR, VPA, or CHIR plus VPA (CHV). Utricles were analyzed 10 days after drug treatment. (**E**) DT-ablated *Pou4f3^DTR/+^*utricles without drug treatment and with drug treatment (+CHIR, +VPA, and +CHV). (**F**) Quantification of MYO7A^+^ cells from in vitro untreated and drug-treated utricles. HC numbers were significantly increased after CHV treatment compared with treatments with CHIR or VPA alone. All data represent the mean ± SEM. **P < 0.01, ****P < 0.0001 by 1-tailed Student’s *t* tests (**C**) and 1-way ANOVA with Tukey’s multiple comparison test (**F**). Scale bar: 50 μm. A, anterior; L, lateral; M, medial; P, posterior.

To ablate HCs, we used *Pou4f3^DTR^* transgenic mice, in which the *Pou4f3* promoter drives the expression of the human diphtheria toxin receptor (DTR) (11). In these mice, diphtheria toxin (DT) selectively ablates cells, including HCs, that express *Pou4f3* (33, 34). We administered DT at day 0 and day 2 (Figure 1A) to *Pou4f3^+/+^* (WT) and *Pou4f3^DTR/+^* heterozygous mice at 4 weeks of age, when HCs are considered mature (35). We assessed HC damage in the utricle, an otolith organ sensitive to linear acceleration, based on HC counts using two separate markers for HCs, POU4F3 and MYO7A.

We observed a 67.7% reduction in HCs by day 4 after DT, with further loss (92%) at day 7 by counting HCs expressing MYO7A (Figure 1, B and C) or Pou4f3 (Supplemental Figure 1, B and C; supplemental material available online with this article; https://doi.org/10.1172/JCI181201DS1). DT treatment in WT mice of the same age did not result in significant HC loss (Supplemental Figure 1D). We thus confirmed that DT treatment induced a selective HC ablation in 4-week-old mouse utricle over a period of 7 days and consistently resulted in near-complete ablation of HCs.

To study the effect of drug treatment on vestibular HC regeneration, utricles from in vivo DT-ablated WT and *Pou4f3^DTR/+^* mice were cultured in the presence or absence of a GSK-3β inhibitor (CHIR), an HDAC inhibitor (VPA), or a combination of the two drugs (CHV). CHV treatment resulted in a significant increase in MYO7A counts at 2, 5, 10 and 14 days after treatment (Supplemental Figure 2). The subsequent studies were performed at day 10 (Figure 1D), where the effect reached a plateau (Supplemental Figure 2E). CHV treatment regenerated 4 times more HCs than DT-ablated organs without drug treatment, which was taken as a measure of spontaneous regeneration in the utricle (Figure 1E). When administered alone, CHIR and VPA were less effective in driving HC differentiation: CHV was 2.5 times more effective that VPA alone and 3.7 times more effective than CHIR alone (Figure 1F). The change in HC number in utricles treated with CHIR alone was not significant.

### Ex vivo regeneration of type I and type II HCs by CHV treatment.

**Figure 2:**
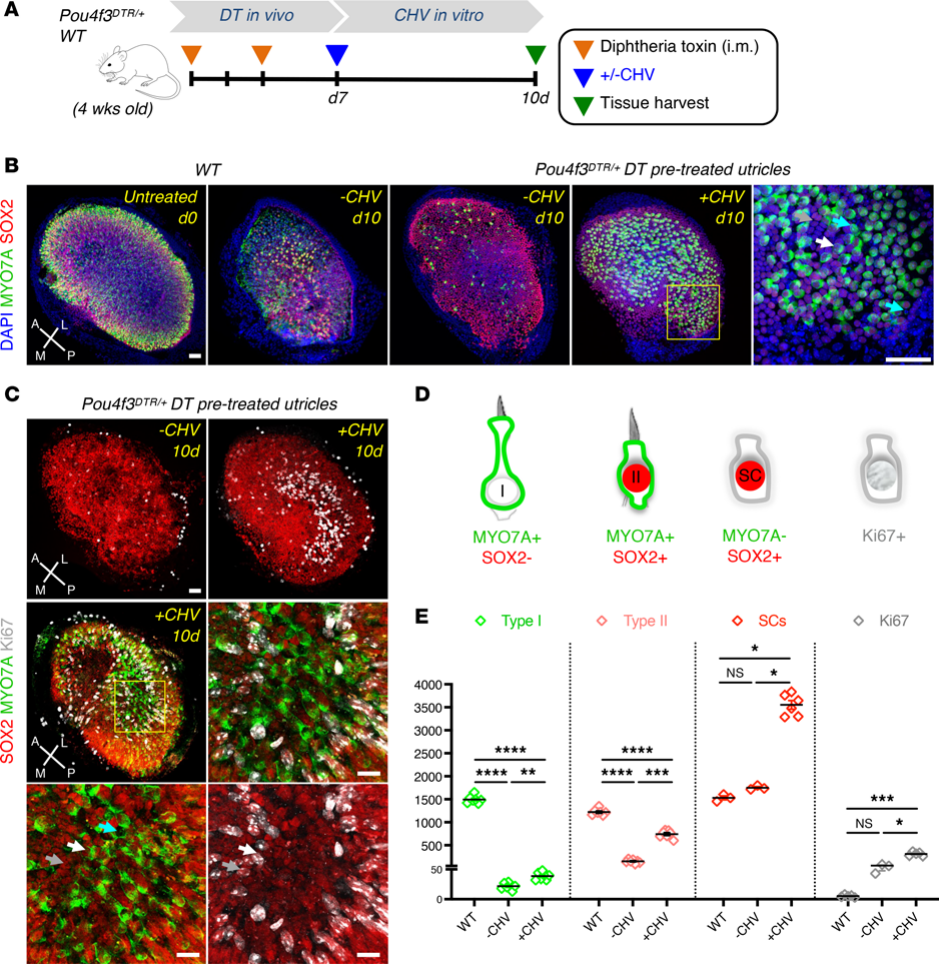
Ex vivo CHV treatment increased SC proliferation and differentiation into type I and type II HCs in DT-ablated *Pou4f3^DTR/+^* mouse utricle. (**A**) Schematic of in vitro drug treatment. At 7 days after damage, utricles were harvested from WT and *Pou4f3^DTR/+^* mice and cultured for 10 days in the absence (–CHV) or presence (+CHV) of drug. (**B**) WT and DT-ablated utricles with or without CHV treatment immunolabeled for MYO7A (green), SOX2 (red), and nuclei stained with DAPI (blue). High-magnification images show the presence of SCs (gray arrow), type I HCs (cyan arrows) and type II HCs (white arrows. (**C**) Ki67 immunostaining (gray) in DT-ablated, untreated, and drug-treated utricles. (**D**) Diagram showing identification strategy for type I HCs, type II HCs, SCs, and newly-divided SCs. (**E**) Quantification of type I and type II HCs, SCs, and Ki67. An increase in SCs and Ki67 counts was accompanied by an increase in type I and type II HC numbers. All data represent the mean ± SEM. *P < 0.05, ***P < 0.001, ****P < 0.0001, by 2-tailed Student’s *t* tests and 1-way ANOVA with Tukey’s multiple comparison test (**E**). Scale bar: 50 μm (**B** and **C**, images showing the whole utricle); 100 μm (**B** and **C**, high magnification images). A, anterior; L, lateral; M, medial; P, posterior.

To further explore the HC regeneration induced by CHV treatment, we focused on identification of type I and type II HCs at day 10 by the differential expression of MYO7A and SOX2 and confirmed these differences with additional markers (Figure 2, A and B). MYO7A labeled all utricular HCs while SOX2 was exclusive to SCs and type II HCs (Figure 2C). As shown in Figure 2D, type I HCs are MYO7A^+^SOX2^–^, type II HCs are MYO7A^+^SOX2^+^, and SCs are MYO7A^–^SOX2^+^. The specificity of SOX2 for type II HCs was confirmed by type II HC marker annexin A4 (ANXA4) (24) labeling of MYO7A^+^SOX2^+^ HCs and type I HC marker secreted phosphoprotein 1 (SPP1, also referred to as osteopontin) (36) labeling of MYO7A^+^SOX2^–^ cells (Supplemental Figure 3). Type I and type II HC counts from SPP1 and ANXA4 were comparable to MYO7A^+^SOX2^–^ and MYO7A^+^SOX2^+^, respectively (Supplemental Figure 3, B and D). HC counts were also consistent with those reported by Desai et al. (37) (Supplemental Figure 3E). After 10 days of CHV treatment, SC and type I HC numbers increased 2-fold, while type II HC counts were 4.5 times higher than untreated organs (Figure 2E). Notably, the increase in SC and HC numbers correlated with increased Ki67 expression, indicating proliferation (Figure 1, C and E).

### Ex vivo lineage tracing of SCs to HCs.

**Figure 3:**
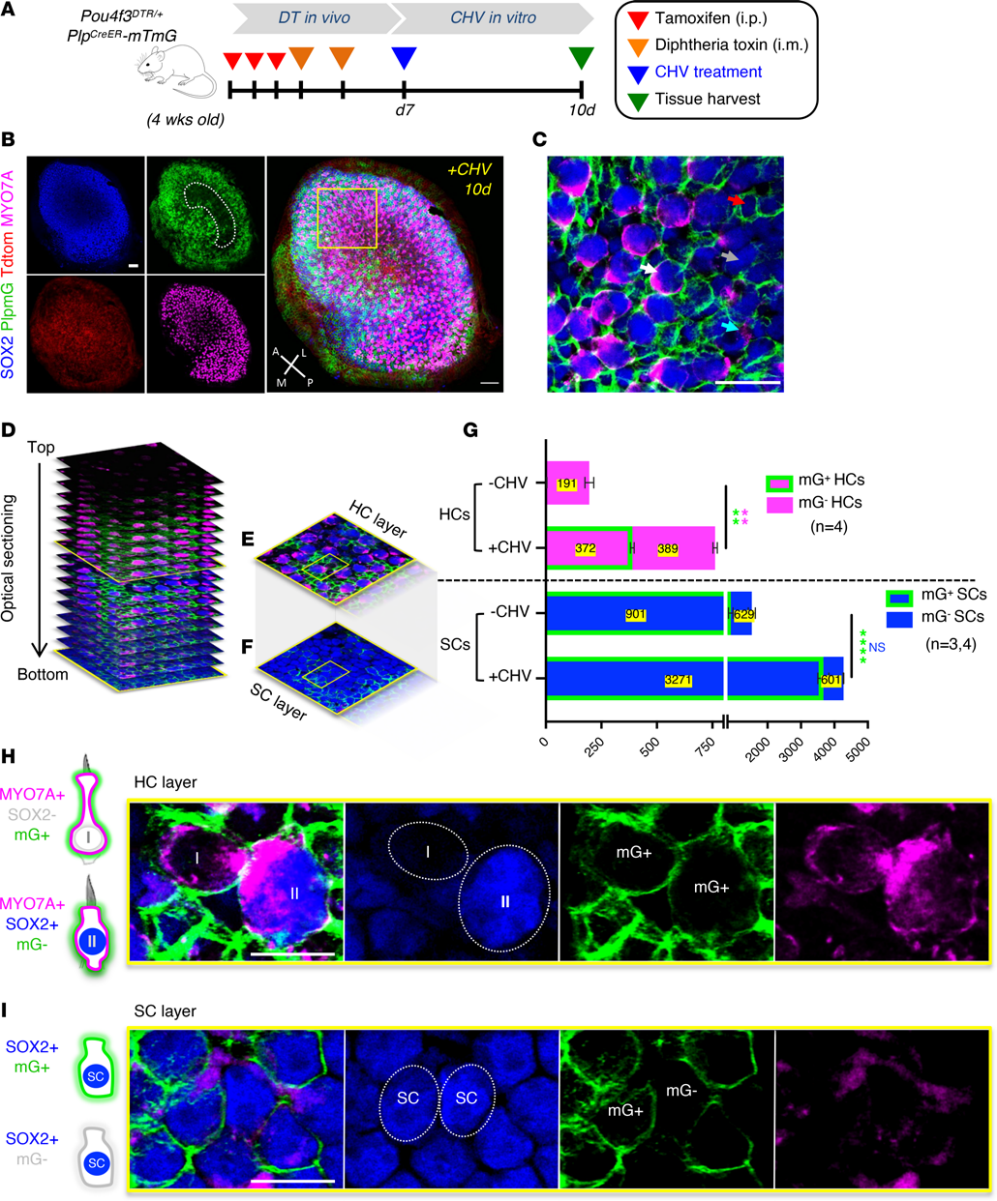
SCs are the primary source of newly regenerated HCs. (**A**) Experimental strategy for fate mapping of SCs and HCs. Utricles from DT-ablated *Pou4f3^DTR/+^-Plp1Cre-mTmG* mice were harvested and cultured in CHV supplemented medium for 10 days. (**B**) Utricles immunolabeled for MYO7A (magenta) and SOX2 (blue). (**C**) High-magnification image indicating Plp mG+ and mG– expressing SCs (red and gray arrows), type I HCs (cyan arrow), and type II HCs (white arrow). (**D**) 3D reconstruction of optical sections showing the mapping of type I and type II HCs. (**E**) HC layer. (**F**) SC layer. (**G**) HC and SC mG+ and mG– counts from CHV-treated and -untreated utricles. (**H**) High-magnification images show a type I HC (I) and a type II HC (II) arising from Plp mG+ SCs (mG+). (**I**) High-magnification images of Plp mG+ and Plp mG– SCs (mG+ and mG–). All data represent the mean ± SEM. ***P < 0.001, ****P < 0.0001, by 2-tailed Student’s *t* test and 2-way ANOVA (**G**). Scale bar: 50 μm (**B**, images showing the whole utricle); 100 μm (**C**, **H**, and **I**). A, anterior; L, lateral; M, medial; P, posterior.

Several lines of evidence have shown that SCs are the principal source of regenerated HCs in the utricle, differentiating into type II but not type I HCs (23, 25, 29). To determine the origin of newly regenerated HCs, we employed a lineage-tracing approach (Figure 3A). We crossed *Pou4f3^DTR/+^* mice with tamoxifen-inducible *Plp1^CreER^*;*mTmG* mice, in which Cre recombinase is expressed under the control of the *Plp1* promoter. Prior to Cre activation, *mTmG* cells exhibit red fluorescence, and, after tamoxifen injection, this switched to green fluorescence in the membrane of *Plp1^CreER^*-expressing (referred to here as mG+) cells. As *Plp* expression is restricted to SCs in the utricular sensory epithelium (38), the *Pou4f3^DTR/+^;Plp1^CreER^*;*mTmG* allows fate mapping of SCs.

Confocal *Z*-series in CHV-treated utricles with the lineage tag exhibited two distinct morphologies (Figure 3, B and C, and Supplemental Video 1): some HCs expressed MYO7A as a ring encircling the nucleus, while others had MYO7A labeling below the nucleus, separating the cells into distinct upper and middle layers characteristic of type I and type II HCs, respectively (3) (Figure 3, C and D). This distribution was consistent with the production of type I and type II HCs identified by MYO7A and SOX2 labeling (Figure 3, E and G). Quantitative analysis of CHV-treated utricles with the lineage tag showed the presence of mG+ and mG– HCs (Figure 3G). HCs were equally divided between mG+ and mG– in CHV-treated utricles, while HCs were mostly mG– in the absence of treatment. mG+ HCs are likely regenerated HCs derived from transdifferentiating Plp1^+^ SCs (Figure 3H), whereas mG– HCs are either cells that remained after damage or regenerated HCs arising from Plp1^–^ SCs present in the striolar region. This is consistent with the *Plp1* promoter inactivity in SCs within the striolar region of mouse utricle (Figure 3B) (18). Of a total of 3,872 SOX2^+^ cells counted at 10 days of CHV treatment, 3,671.4 ± 272.6 were mG+ and 601.5 ± 11.6 were mG– (Figure 3, F and I). These SOX2^+^mG– counts are comparable to the total number of SCs in the striolar region of mouse utricular sensory epithelium (535 ± 18 SCs) (37).

### Restoration of vestibular function after CHV treatment.

**Figure 4:**
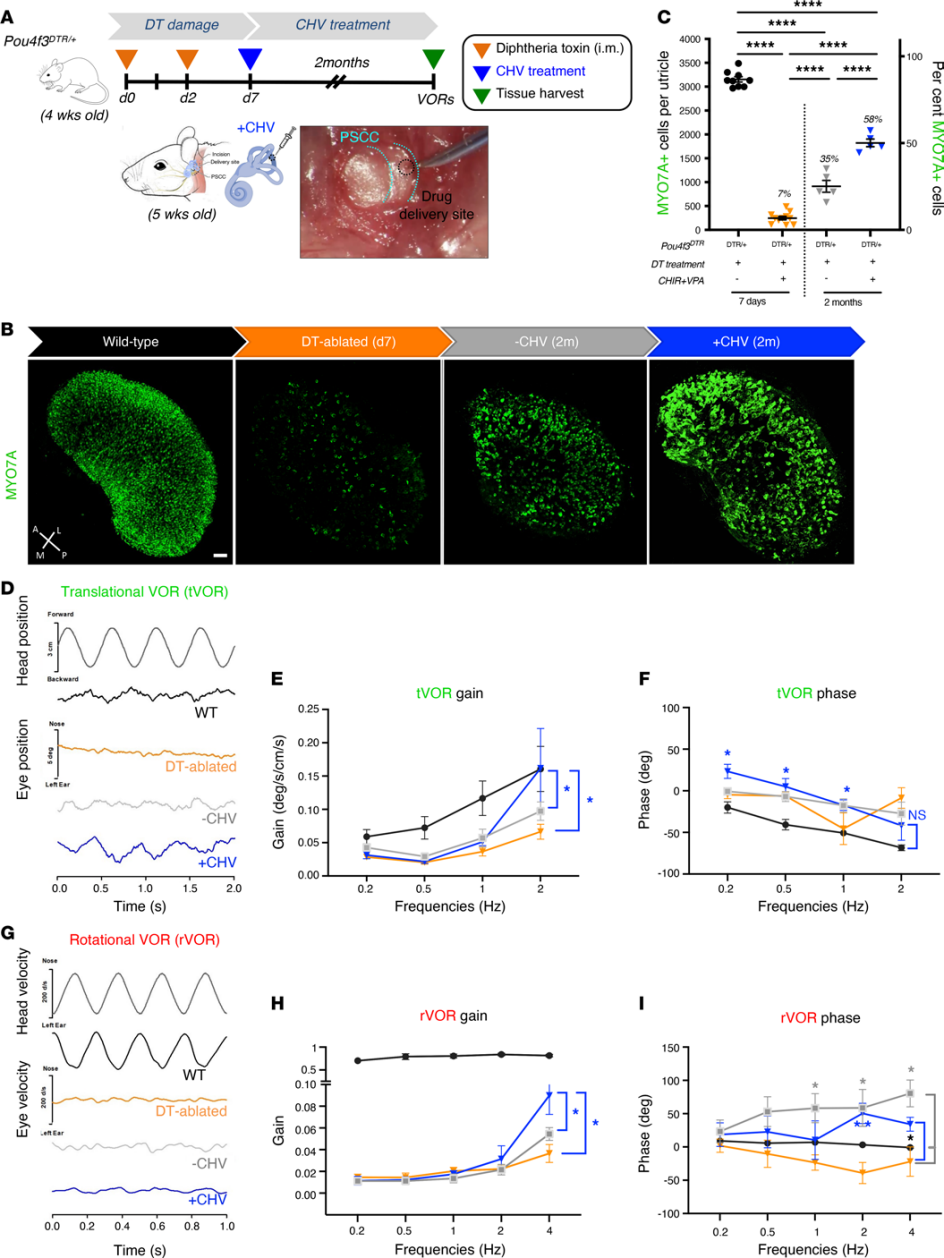
In vivo CHV treatment improves vestibular function by promoting HC regeneration. (**A**) Schematic of the in vivo approach for drug delivery in DT-ablated *Pou4f3^DTR/+^* mice. CHIR and VPA were injected in the left ear via the posterior semicircular canal (PSCC) at day 7 after DT ablation; the contralateral ear was used as a control for spontaneous regeneration. Mice were examined at 2 months after drug treatment. (**B**) MYO7A immunolabeled utricles from 2-month WT, DT-ablated, untreated (–CHV), and treated (+CHV) ears. (**C**) MYO7A counts from WT utricles (black), DT-ablated utricles (orange), DT-ablated utricles without CHV treatment (gray), and DT-ablated utricles with CHV treatment (blue). (**D**) Translational vestibuloocular reflexes (tVORs) in response to sinusoidal head translations at 2 Hz for WT (black), DT-ablated (orange), and DT-ablated mice with (blue) and without (gray) CHV treatment. (**E**) tVOR gains. (**F**) tVOR phases. (**G**) Rotational vestibuloocular reflexes (rVORs) to sinusoidal head rotations with representative eye velocity responses to 4 Hz head rotation. (**H**) rVOR gains. (**I**) rVOR phases. Numbers of mice tested for each group were 3 for WT (black), 8 for DT ablated (orange), 8 for DT-ablated without CHV treatment (gray), and 5 for DT-ablated with CHV treatment (blue). All data represent the mean ± SEM. ****P < 0.0001 by 1-way ANOVA (**C**), *P < 0.05, by 2-tailed Student’s *t* tests (**E** and **H**), and *P < 0.05, **P < 0.01 by 2-way ANOVA with Tukey’s multiple comparison test (**F** and **I**). Scale bar: 50 μm. A, anterior; L, lateral; M, medial; P, posterior.

To investigate whether utricular HC differentiation could be induced in mature vestibular organs in vivo, we administered CHV to DT-ablated *Pou4f3^DTR/+^* mice starting at day 7 after HC ablation by unilateral injection into the posterior semicircular canal (PSCC) (Figure 4A). After 1 month (Supplemental Figure 4A), both untreated (Supplemental Figure 4B) and drug treated (Supplemental Figure 4, C and E) ears showed a significant increase in MYO7A^+^ cells. However, HC regeneration was significantly higher in the CHV-treated ears compared with the untreated ears (Supplemental Figure 4D); this increase was associated with sustained proliferation (Supplemental Figure 5). No further change in HC number was observed in the untreated ears at 2 months, while a further increase in HCs was apparent in CHV-treated ears, with 58% of HCs replaced (Figure 4, B and C).

Consistent with the near-complete HC ablation (92%), translational and rotational VORs (tVOR and rVOR), which allow gaze stabilization in response to head translation and rotation, were absent in the DT-ablated mice (Figure 4, D and G). WT mice exhibited high compensatory tVOR and rVOR at all frequencies. The substantial regeneration of HCs in the CHV-treated utricles at 2 months resulted in restoration of tVOR at 2 Hz. The mice exhibited compensatory gains and phases comparable to WT levels (Figure 4, E and F). The rVOR did not exhibit similar recovery, although a small, statistically significant increase in rVOR gain was observed in the CHV treated mice at 4 Hz (Figure 4, H and I).

### Restoration of vestibular afferent activity.

**Figure 5:**
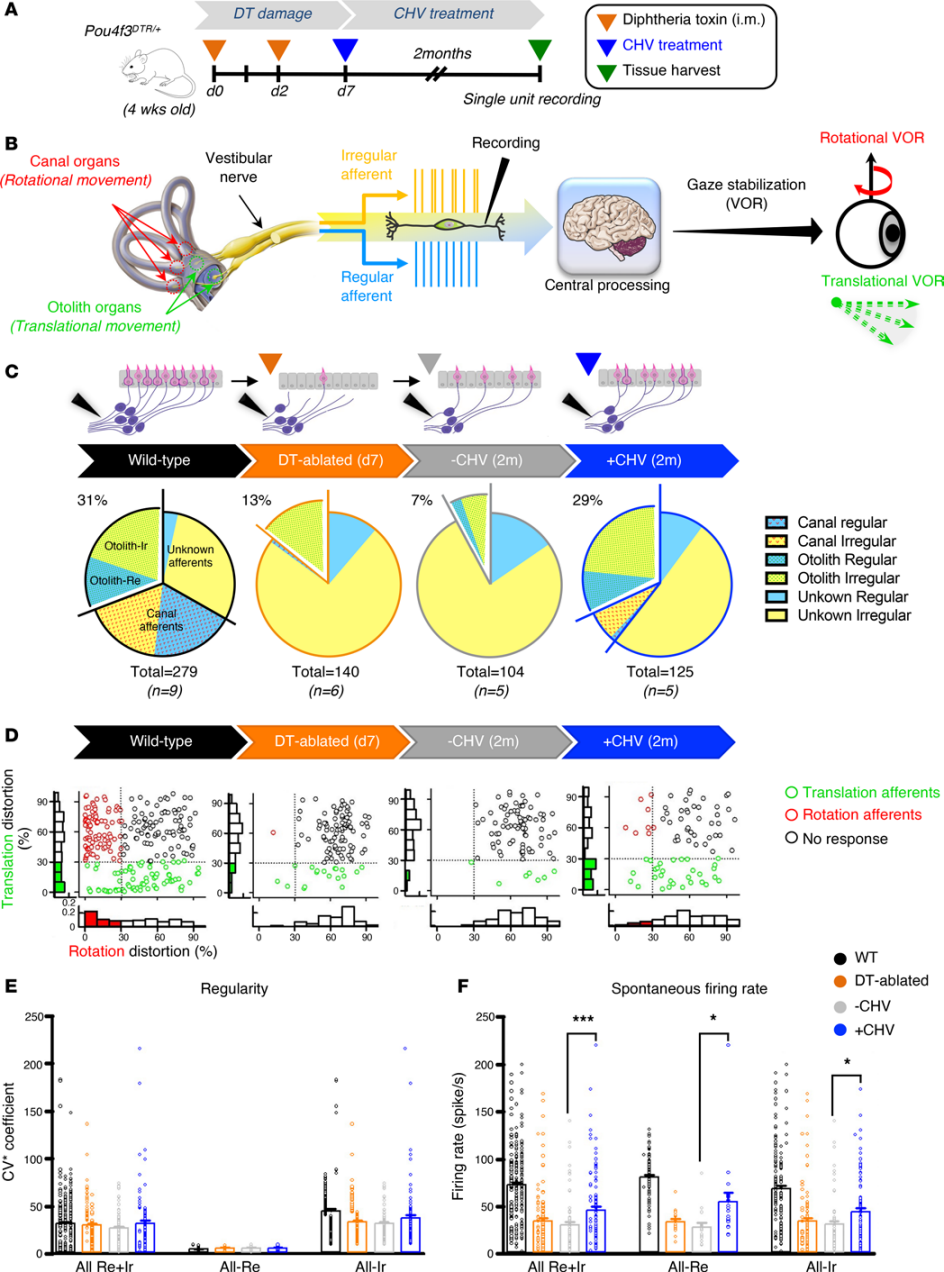
CHV treatment restores vestibular afferent activity in DT-ablated mice. (**A**) Schematic depicting the experimental approach. Four-week-old WT and *Pou4f3^DTR/+^* mice were injected with DT at day 0 and day 2, followed by a local injection of CHV via the semicircular canal at day 7. Single unit recordings of the vestibular nerve were performed 2 months after drug treatment. (**B**) Schematic showing response dynamics of vestibular afferents from sensory epithelia. Afferents receive inputs from HCs through regular and irregular channels. Each canal afferent encodes information about angular head motion while otolith afferents encode information about translational acceleration in response to gravity. (**C**) Vestibular afferents recorded from the vestibular nerves of 9 WT mice (*n* = 279 afferents), 6 DT-ablated mice (*n* = 140 afferents), 5 DT-ablated mice without CHV (–CHV, *n* = 104 afferents), and 5 DT-ablated mice with CHV (+CHV, *n* = 125 afferents) mice. (**D**) Comparative analysis of afferent distribution. The histograms in the *x* axis represent rotation distortion, and the *y* axis represents translation distortion. (**E**) Vestibular afferent regularity. Normalized coefficient of variation (CV*) of interspike intervals from WT mice (black), DT-ablated mice (orange), and DT-ablated mice with (blue) and without (gray) CHV treatment. (**F**) Averaged spontaneous firing rates from the same groups. All data represent the mean ± SEM. *P < 0.05, ***P < 0.001, by 1-tailed Student’s *t* tests (**F**). A, anterior; L, lateral; M, medial; P, posterior.

To assess the extent of recovery of vestibular afferent signals, recordings from single neurons were performed from otolith and canal organs (Figure 5A). Single units were recorded from a total of 648 afferents from the vestibular nerve bundles of 25 mice: 9 WT mice, 6 DT-ablated mice, 5 mice without CHV treatment, and 5 mice with CHV treatment (Figure 5, B and C). Afferents were classified as otolith or canal based on their response to head translation and rotation and as regular or irregular based on their normalized coefficient of variation of interspike intervals (CV*).

The precision of vestibular afferents in encoding head motion was quantified using a distortion metric. The distortion revealed that CHV treatment substantially enhanced the afferents’ ability to accurately transduce translational and angular stimuli into neural signals (Figure 5D). The regularity of neuronal firing across groups was comparable to WT (Figure 5E), suggesting that the consistency of vestibular function is maintained despite HC loss and subsequent replacement. The spontaneous firing rate of both regular and irregular afferents was significantly increased in drug-treated mice compared with the untreated group (Figure 5F). Twenty-nine percent of otolith afferents were responsive to head translation (Supplemental Figure 6), which is close to the number in WT mice, while only approximately 7% of afferents were responsive to head rotation in the CHV group (Supplemental Figure 7), indicating differential effects of the drug treatment on the canal and otolith organs. These changes align with the increased gain in both tVOR and rVOR after the significant (92%) loss of HCs.

**Figure 6:**
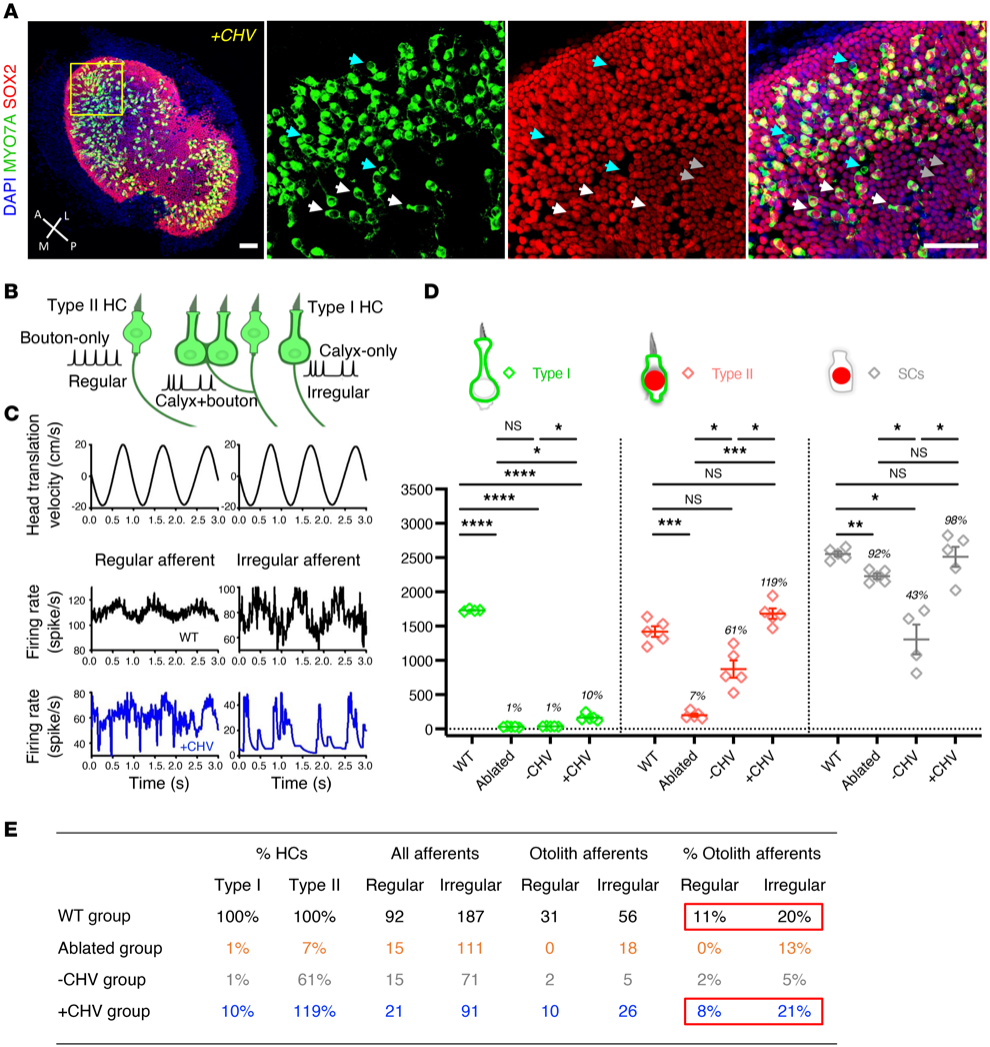
Recovery of translation-evoked otolith afferent responses and replacement of type I and type II HCs by CHV treatment. (**A**) *Pou4f3^DTR/+^* DT-ablated utricles, 2 months after drug treatment, immunolabeled for MYO7A (green) and SOX2 (red). Cell nuclei were stained with DAPI (blue). High-magnification images indicate the presence of SCs (gray arrows), type I HCs (cyan arrows), and type II HCs (white arrows). (**B**) Schematic of regular and irregular endings on type I and type II HCs. (**C**) Regular and irregular otolith afferent responses to nasal-occipital head translation in WT (black) and CHV-treated (blue) mice, depicted with red boxes in **E**. (**D**) Quantification of SCs and type I and type II HCs with and without CHV treatment compared with WT and DT-ablated mice 2 months after treatment. (**E**) Type I and type II HCs and regular and irregular otolith afferent responses. The percentages of regenerated HCs compared with regular and irregular afferents in the WT, DT-ablated (7 days), DT-ablated and untreated (2 months), and DT-ablated and CHV-treated (2 months) ears. Single-unit recordings of vestibular afferents are normalized by the total recorded afferents in each group. The percentage of otolith afferents in WT and +CHV groups are boxed in red. All data represent the mean ± SEM. *P < 0.05, **P < 0.01,***P < 0.001, ****P < 0.0001, by 2-tailed Student’s *t* tests and 1-way ANOVA by Tukey’s multiple comparison test (**D**). Scale bar: 50 μm (**A**, image showing the whole utricle); 100 μm (**A**, high magnification image). A, anterior; L, lateral; M, medial; P, posterior

HCs are critical for transducing vestibular signals to be transmitted to the brain by vestibular afferents, thereby allowing gaze stabilization and maintenance of balance. Increased numbers of type I and type II HCs were observed with CHV treatment (Figure 6A). Because regular afferents received inputs from type II HCs only, while irregular afferents received inputs from both type I and type II HCs (Figure 6B), and since CHV restored both regular and irregular afferent activity (Figure 6C), these data are consistent with partial recovery of afferents after replacement of type I and type II HCs. Regenerated HCs after CHV treatment accounted, respectively, for 10% and 119% of WT type I and type II HCs (Figure 6D and Supplemental Table 2). In contrast, spontaneously regenerated HCs were exclusively type II, representing only 61% of the total HCs (Figure 6E). SC numbers after CHV treatment were comparable to WT mice, while untreated ears exhibited a 50% decrease in SC numbers due to spontaneous differentiation. Thus, a single in vivo administration of CHV resulted in a complete replacement of type II HCs with a partial replacement of type I HCs, and newly regenerated HCs reestablished neural connections, leading to improved vestibular function in DT-ablated mice.

## Discussion

The recognition that nonmammalian vertebrates had an inherent capacity to regenerate (39, 40) the mechanosensory receptor cells for hearing and balance initiated a field of inquiry into the blocks to sensory HC regeneration in mammals. HC regeneration has become an important field of study in the mammalian nervous system where sensory cells and neurons in general do not regenerate. A lack of treatments to achieve reversal is a persistent problem for balance disorders, where pharmacological treatments largely consist of drugs to alleviate symptomatic burden without resolving the pathology (2). We demonstrate here that simultaneous inhibition of GSK-3β and HDAC results in an increase in SCs due to proliferation and up to 58% regeneration of HCs in mouse utricle, replacing 10% of type I HCs and all type II HCs. CHV treatment partially restored the afferent innervation to HCs, resulting in substantial recovery of vestibular function.

The work here shows that reprogrammed cells, unlike reprogrammed glia that can give rise to cells with some of the anatomical attributes of neurons (41, 42), can also develop mature function. The CHV treatment may have induced changes in chromatin structure and accessibility that led to the activation of genes required for HC regeneration. The sustained increases in regenerated HCs observed over 2 months may result from the extended time required to initiate epigenetic and chromatin remodeling processes leading to proliferation and differentiation of SCs.

VOR and single-unit recordings showed that the CHV treatment restored spontaneous activity of both regular and irregular afferents, consistent with extensive regeneration of both type I and type II vestibular HCs. Response properties of vestibular neurons can be divided into regular afferents that receive inputs from type II HCs and are responsive to fine changes in velocity and irregular afferents that receive inputs from type I HCs, alone or in combination with type II HCs, and are responsive to positional changes at high frequencies (4, 5, 7). Regular and irregular afferents were partially restored in drug-treated mice. We showed an extensive regeneration of vestibular HCs, particularly type II, and a partial restoration of vestibular function by a clinically relevant metric (VOR) and by direct measurement of movement-induced activity in vestibular neurons. The regeneration of mouse vestibular HCs was achieved here in a damage model in which 92% of HCs are ablated at 4 weeks of age, an age when HCs are mature and there is limited spontaneous regeneration. In contrast with the neonatal utricle, where spontaneously regenerated HCs displayed type I and type II features (23, 29), regenerated HCs in the adult in the same damage model have previously been limited to a type II phenotype (11, 25, 26), and the number of regenerated HCs has been minimal (10, 12). The recovery of VOR gain seen here in adult mice treated with CHV was not observed in damaged but untreated animals, and the regeneration of HCs (58% of the WT level) was nearly twice that of spontaneous regeneration. An approach using *Atoh1* overexpression to augment HC production restored type II but not type I HCs after P30 HC ablation, but the improvement was only slightly greater than the spontaneous recovery in this damage model, which had a lower degree of HC death than ours, and improvements in vestibular evoked potentials were variable (20). Moreover, constitutively expressed *Atoh1* could have a deleterious effect on HCs as noted elsewhere (43, 44). Here, all animals treated with CHV showed a recovery in VOR at high frequency. Although the focus of the study was on HC regeneration in the utricle, the effects of DT treatment and CHV treatment on canal function were assessed by single-unit recording and rVOR. In contrast to the robust recovery of otolith afferent responses to translation and the tVOR, recovery of the rVOR and canal afferent responses to rotation were limited. The rotation responsive afferents showed a modest increase (from 0% in the spontaneous regeneration group to 7% in the CHV treatment group), and rVOR gain was slightly increased by CHV treatment.

We discovered previously that a drug cocktail consisting of a GSK-3β inhibitor to activate Wnt signaling and an HDAC inhibitor to modify the openness of chromatin increased proliferation of SCs and differentiation of HCs in organoids made from Lgr5-expressing cochlear progenitor cells (45). Lgr5^+^ cells in the cochlea act as progenitors that account for HC production in the newborn cochlea stimulated by activation of Wnt (46, 47), and we have recently demonstrated by single cell gene expression analysis (48) the gene regulatory network through which the cochlear progenitors treated with these two drugs differentiate to HCs. Other work has shown that Atoh1 is a target of Wnt signaling and that the limited HC regeneration recently observed in newborn mammalian cochlea is dependent on Wnt signaling (46, 49–51). Lgr5 potentiates the activity of Wnt in numerous cell types and was also expressed in the newborn utricle after damage, where the cells expressing Lgr5 gave rise to type I and type II HCs (23, 29).

However, the epigenetic landscape of postnatal SCs comprises a block to regeneration that increases with age, and this block is seen for Wnt signaling and Atoh1 activation (47, 52). The epigenetic status of the vestibular system is in general more permissive for regeneration than the cochlea (53). Mammalian vestibular HCs regenerate spontaneously early in life (19), but the extent of the regeneration decreases with age (11, 12, 25, 54), potentially explaining the need for epigenetic manipulation to overcome resistance to the effects of Wnt signaling. Increased SC proliferation and differentiation to HCs when CHIR and VPA were combined, compared with use as single agents, was consistent with this hypothesis.

The regeneration of HCs is an important challenge for clinical progress in the treatment of balance disorders. Our success in overcoming the resistance to regeneration is obviously important for translation, as the loss of vestibular function progresses with age to become extremely common in older adults (1, 2). Patients with balance disorders experience severe debilitation and social burden; this impairment is attributed in many cases to degeneration of HCs. The drug combination described here is thus a step toward the development of a regenerative therapy for the restoration of vestibular function.

## Methods

### Sex as a biological variable.

In the present studies, mice of both sexes were used in all in vivo and in vitro experiments.

### Mice.

All mice were obtained from The Jackson Laboratory and were housed with open access to food and water. *Pou4f3^DTR^* mice (JAX, 028673) (11) were mated with C57BL/6 mice to obtain *Pou4f3*^DTR/+^ mice for HC ablation, and *Pou4f3^+/+^* (WT) mice were used as controls. For lineage-tracing experiments, we used *Plp1^CreER^* reporter lines crossed with *mTmG*. Littermates were genotyped using protocols from The Jackson Laboratory (Supplemental Table 1) and mated with *Pou4f3^DTR/+^* mice to obtain *Pou4f3^DTR/+^;Plp1^CreER^*;*mTmG* mice. The mice were shipped to University of Mississippi Medical Center for VOR and single vestibular fiber recordings. Recordings were processed by laboratory staff blinded to VOR performance and vestibular function testing.

### DT administration.

DT (MilliporeSigma, D0564) was reconstituted in distilled water at 1 mg/mL as a stock solution and stored at –20°C. A working solution was diluted in sterile PBS 1× at 10 μg/mL and freshly prepared prior to each injection. Four-week-old mice received 2 intramuscular injections of DT at 50 ng/g, spaced 2 days apart. Mice were hydrated with 0.4 mL of lactated Ringer’s solution injected subcutaneously at day 0, day 1, and day 2. Mice were fed a high-calorie diet, and hydrogel was provided in the cage.

### Tamoxifen injections.

For Cre activation, tamoxifen (MilliporeSigma, T5648) was dissolved in corn oil at 50 mg/mL and administered by intraperitoneal injection to *Pou4f3^DTR/+^;Plp1^CreER^*;*mTmG* mice at P25 for 3 consecutive days prior to DT treatment (2.5 μL/g of tamoxifen solution).

### Whole-organ culture.

Utricles were harvested from 5-week-old mice pretreated with DT, as described above. Utricles of both mice of both sexes were dissected in cold DMEM/F12 under sterile conditions. After removing the otoconia, utricles were plated in 4-well petri dishes (1 utricle per well) prefilled with 90 μL growth factor-enriched medium, consisting of DMEM/F12 (1:1), N2 (1:100), and B27 (1:50) and incubated at 37°C. One hour later, medium was replaced and supplemented with CHIR (3 μM), VPA (1 mM), CHV, or 0.1% DMSO (controls). Whole organs were maintained in an incubator (37°C, 5% CO_2_) with medium changes every day, and subjected to immunohistochemical analysis after 2, 5, 10 and 14 days.

### Surgical approach.

Seven days after DT treatment, 5-week-old mice of both sexes received a unilateral injection of drug (CHV) via the PSCC; the contralateral ear was used as a control. CHIR and VPA were diluted in polyethylene glycol 400 (1:2) and artificial perilymph (1:2) to a final concentration of 5.3 mM and 300 mM, respectively. A micro syringe pump was used for drug delivery.

Before treatment with CHIR and VPA, mice were anesthetized with an intraperitoneal injection of ketamine (100 mg/kg) and xylazine (10 mg/kg). All the surgical procedures were performed in prewarmed room (26°C) to maintain the body temperature. The fur behind the left ear was shaved with a razor and sterilized with 10% povidone iodine. Under an operating microscope, a postauricular incision was made to access the temporal bone, and the facial nerve was identified along the wall of the external auditory canal. After exposing the facial nerve and the sternocleidomastoid muscle by blunt dissection, a portion of the muscle was divided using an electrosurgical cutter. To visualize the PSCC, the muscles covering the temporal bone were separated dorsally using retractors. A small hole was gently made in the PSCC using an insulin syringe. After 5 minutes for leakage of perilymph to abate, the tip of the polyimide tube was inserted into the canal. The aperture between the polyimide tube and the hole was sealed with muscle fragments and cyanoacrylate glue; the tightness of sealing was visually assessed by the lack of fluid leakage. When the glue dried, 500 nL of drug suspension was injected at a speed of 91 nL/minute. The tube was then cut and plugged with small pieces of muscle, and the skin was closed with 4-0 nylon sutures. Total surgical time ranged from 50 to 60 minutes per mouse. Hydrogel and high-caloric food gel were placed into the cage daily from the first day after drug treatment until recovery. Pain was controlled with buprenorphine (0.05 mg/kg) given directly postoperatively and meloxicam (2 mg/kg) injected every 20–24 hours for 3 days. Recovery was closely monitored daily for at least 5 days postoperatively.

### VOR.

Detailed methods were previously described (55–57). Briefly, each mouse was implanted with a head holder on the skull. Eye position signals of the left eye were recorded using an eye tracking system (ISCAN, ETS-200) that was mounted on a servo-controlled rotator/sled (Neurokinetic). The eye tracker tracked the pupil center and a reference corneal reflection at a speed of 240 frames per second with a spatial resolution of 0.1 degree. Calibration was achieved by rotating the camera from the left 10 degree to the right 10 degree around the vertical axis of the eye. Following calibration, horizontal head rotations were delivered at 0.2, 0.5, 1, 2, and 4 Hz (60 degree/s peak velocity) to measure the steady-state rotational VOR responses. Signals related to eye position and head position were sampled at 1 kHz at 16-bits resolution by a CED Power 1401 system (Cambridge Electronics Devices). Eye movement responses were analyzed using Spike2 (Cambridge Electronics Devices), MatLab (MathWorks), and SigmaPlot (Systat Software). Eye position signals were filtered and differentiated with a bandpass of DC to 50 Hz to obtain eye velocity. Gains and phases of the rotational VORs were calculated by performing a fast Fourier transform on the desaccaded eye velocity signal and head rotation velocity signal as described previously (57). The VORs were tested in a deceased mouse across all frequencies to ensure that the observed responses were not artifacts (56).

### Vestibular afferent recording.

Single-unit recording of vestibular afferents was performed under ketamine/xylazine anesthesia as described previously (57–59). The head was stabilized on a stereotaxic frame (David Kopf Instruments) via the head holder. The animals’ core body temperature was monitored and maintained at 36°C–37°C with a heating pad (Frederick Haer & Company, Bowdoinham). A craniotomy was performed to allow access of the vestibular nerve by a microelectrode filled with 3 M NaCl (40~60 MΩ) (Sutter Instruments). Extracellular recording was performed using a MNAP system (Plexon Inc.). Every spontaneously active nerve fiber encountered was tested. Each afferent’s spontaneous activity was first recorded to calculate the regularity and baseline firing rate. Each semicircular canal was then brought into the plane of earth-horizontal rotation, and the isolated afferent’s responses to head rotation along with horizontal and vertical head position signals were recorded. Extracellular voltage signals were sampled by a CED at 20 kHz with 16-bit resolution and a temporal resolution of 0.01 ms. Head position signals were sampled at 1 kHz. Regularity of vestibular afferents was determined by calculating their normalized CV*s. Vestibular afferents were classified as regular (CV* ≤ 0.1) or irregular (CV* > 0.1) units based on their CV* (6, 60, 61). To quantify an afferent’s responses to head rotation, the fundamental response was extracted from the averaged data using a fast Fourier transform analysis. Gains and phases relative to head velocity were calculated at 1 Hz. Given that many afferents in damaged mice showed minimal modulation during head movement, traditional gain metrics were insufficient for assessing signal significance in their discharge activities. To address this, we employed a distortion metric, defined as 1 – (amplitude of the fundamental response)/(square root of the sum of the squared amplitudes of the first 10 harmonics), for a more statistically confident evaluation of head movement signals. This distortion metric, akin to “stimulus-response coherence,” is particularly effective for assessing vestibular afferent responses to head rotation and translation in animal models with damaged vestibular end organs resulting from genetic mutations, trauma, and ototoxicity (62). An afferent with translation distortion ≤30% is classified as a translation/otolith afferent. An afferent with rotation distortion ≤30% and translation distortion >30% is classified as a rotation/canal afferent. An afferent with rotation distortion >30% and translation distortion >30% is classified as a no response afferent.

### Immunohistochemistry.

Utricles were harvested and fixed for 30 minutes in 4% paraformaldehyde in PBS, pH 7.4 (Electron Microscopy Services) at room temperature. Unspecific binding was blocked with 10% donkey serum, 0.25% Triton X-100 in PBS for 1 hour at room temperature. Tissues were incubated overnight at 4°C with specific primary antibodies diluted in blocking solution (Supplemental Table 1). Then utricles were rinsed (3 times for 5 minutes each) in PBS and incubated with the corresponding Alexa Fluor secondary antibodies (2 hours at room temperature) (Supplemental Table 1), and nuclei were counterstained with DAPI (1:1,000). Samples were mounted using Prolong Gold antifading (Life Technologies) on glass slides. Positive and negative controls were performed for all immunostaining. The images were acquired with Leica SP8 confocal microscopy and analyzed with Fiji-ImageJ.

### Cellular quantification.

Cells were counted manually using graphic tools, cell counter plugins, of Fiji-ImageJ software. Total HC and SC counts per utricle were performed from 20× *Z*-stack images of 29,790 μm^2^ using a grid method or from the whole sensory epithelium for the newly regenerated HCs. The count from 29,790 μm^2^ represents the quantification in 15 grids (1,986 μm^2^ each) randomly chosen from both striolar and extrastriolar regions that fell within the sensory epithelium. To get the total cell number per utricle, we computed the area of the sensory epithelium using ImageJ-Measure. We then multiplied the total area by the summed counts from 15 grids and divided the result by the counted area (29,790 μm^2^). Only cells that had a healthy-appearing DAPI-labeled nucleus located in the sensory epithelium were counted as positive.

### Statistics.

The statistical analyses for each figure are indicated in the figure legends and include 1-way and 2-way ANOVA, 1-way ANOVA and 2-way ANOVA with Tukey’s multiple comparison tests, and 1-tailed and 2-tailed Student’s *t* tests. All statistical analyses were performed using GraphPad Prism Software version 10 for MacOS. All data are presented as mean ± SEM. Two-tailed Student’s *t* tests (or as otherwise indicated) were used to compare means between groups. *P* < 0.05 was considered significant.

### Study approval.

All mouse experiments were approved by the Institutional Animal Care and Use Committee of Massachusetts Eye and Ear and the University of Mississippi Medical Center.

### Data availability.

All study data are included in this article, supplemental figures and tables, and the Supporting Data Values file.

## Author contributions

Conceptualization was provided by AE and HL. Methodology was provided by ASBE, HL, HZ, and WZ. Investigation was provided by ASBE, HL, HZ, and WZ. Visualization was provided by ASBE, HL, HZ, and WZ. Funding was acquired by ASBE. Project administration was provided by ASBE. Supervision was provided by ASBE and HL. The original draft of the manuscript was written by ASBE and HL. The manuscript was reviewed and edited by ASBE, HL, HZ, and WZ.

## Supplementary Material

Supplemental data

Supporting data values

## Version 1 09/24/2024

In-Press Preview

## Version 2 11/15/2024

Electronic publication
