# Supplementary material for: Pharmacological regeneration of sensory hair cells restores afferent innervation and vestibular function
Source: J Clin Invest. 2024 Sep 24;134(22):e181201. doi: 10.1172/JCI181201 (PMC11563683; doi:10.1172/JCI181201)
Supplement: Supplemental data [file jci-134-181201-s194.pdf]

Supplemental Figures

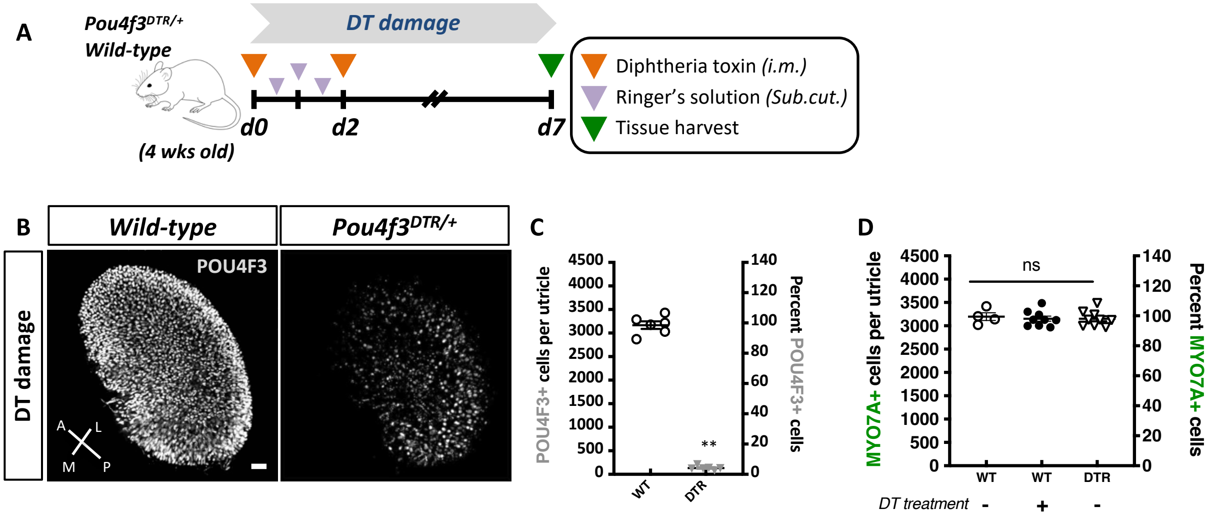

Supplemental Figure 1. HC damage at 4 and 7 days after DT treatment. Related to Figure 1. (**A**) Schematic of DT in vivo damage. 4-week-old *Pou4f3*$^{+/+}$ (WT) and *Pou4f3*$^{DTR/+}$mice received two intramuscular injections of diphtheria toxin (DT). Utricles were analyzed 7 days after damage. **(B)** 5-week-old *Pou4f3*$^{+/+}$ (WT) and *Pou4f3*$^{DTR/+}$ utricles immunolabeled for POU4F3 (gray). **(C)** Pou4f3+ cell counts of WT and DT-ablated utricles. **(D)** HC numbers in *Pou4f3*$^{DTR/+}$ mice were comparable to WT untreated and DT-ablated mice. All data represent the mean ± SEM. $^{**}p < 0.01$ by 1-tailed Student *t* tests (**C**) and 1-way ANOVA with Tukey's multiple comparison test (**D**). Scale bar = 50 μm.

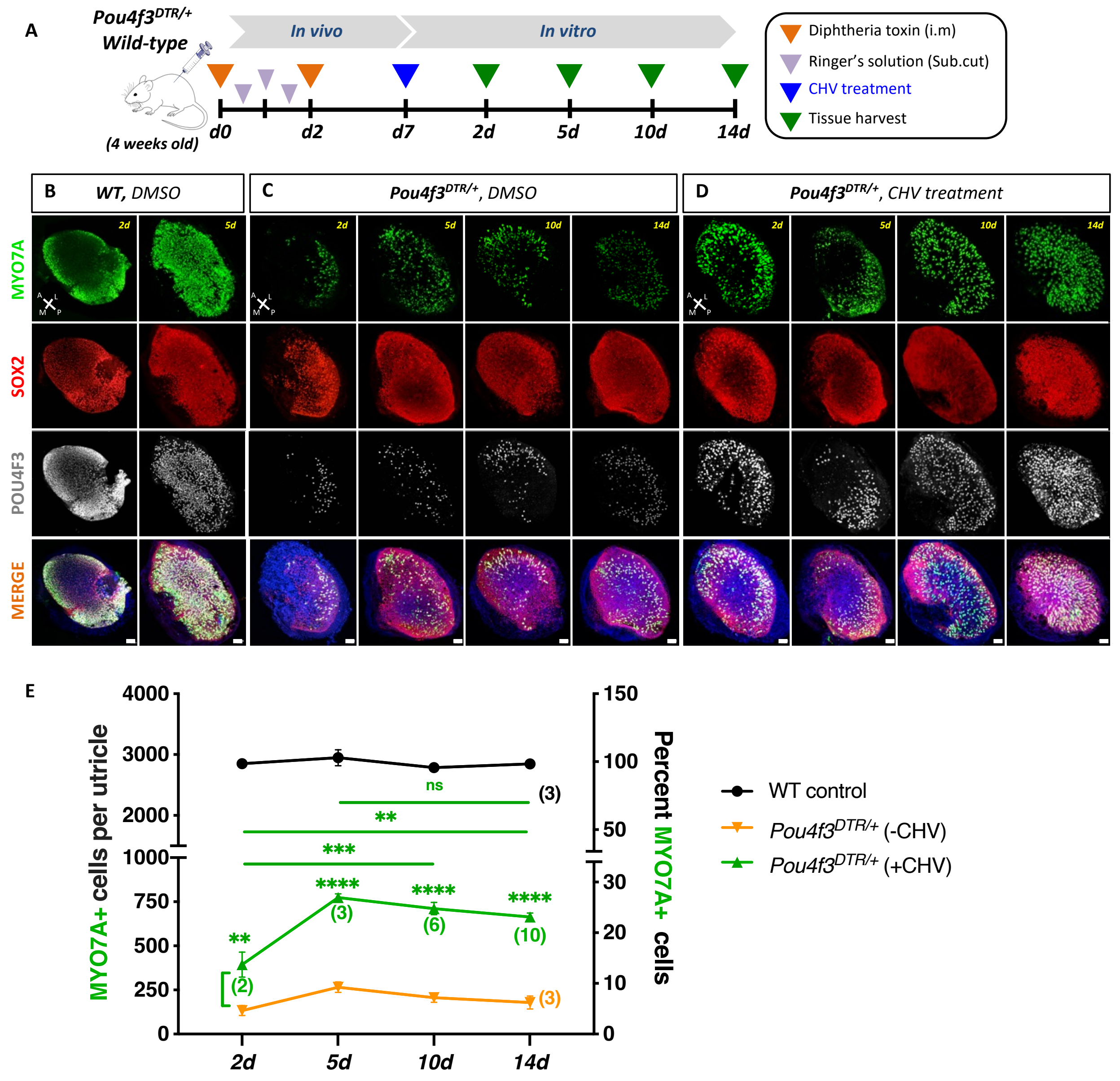

Supplemental Figure 2. In vitro untreated and drug treated utricles. Related to Figure 1. (A) Schematic of the in vitro drug treatment approach. 5-week-old utricles harvested from in vivo DT-ablated, WT, and *Pou4f3*$^{DTR/+}$ mice, were cultured in medium supplemented with either CHV or DMSO (control). Utricles were collected at 2, 5, 10 and 14 days and immunolabelled for MYO7A (green), SOX2 (red) and POU4F3 (gray); cell nuclei were stained with DAPI (blue). (**B**) WT untreated utricles. (**C**) *Pou4f3*$^{DTR/+}$ untreated utricles. (**D**) *Pou4f3*$^{DTR/+}$ CHV treated utricles. (**E**) Quantification of MYO7A immunopositive HCs from WT (black) and DT-ablated utricles, with (blue) and without (orange) CHV treatment. All data represent the mean ± SEM. ****$p < 0.0001$, ***$p < 0.001$, **$p < 0.01$ by 2-tailed Student's t tests and 1-way ANOVA with Tukey's multiple comparison test (**E**). Scale bars = 50 µm.

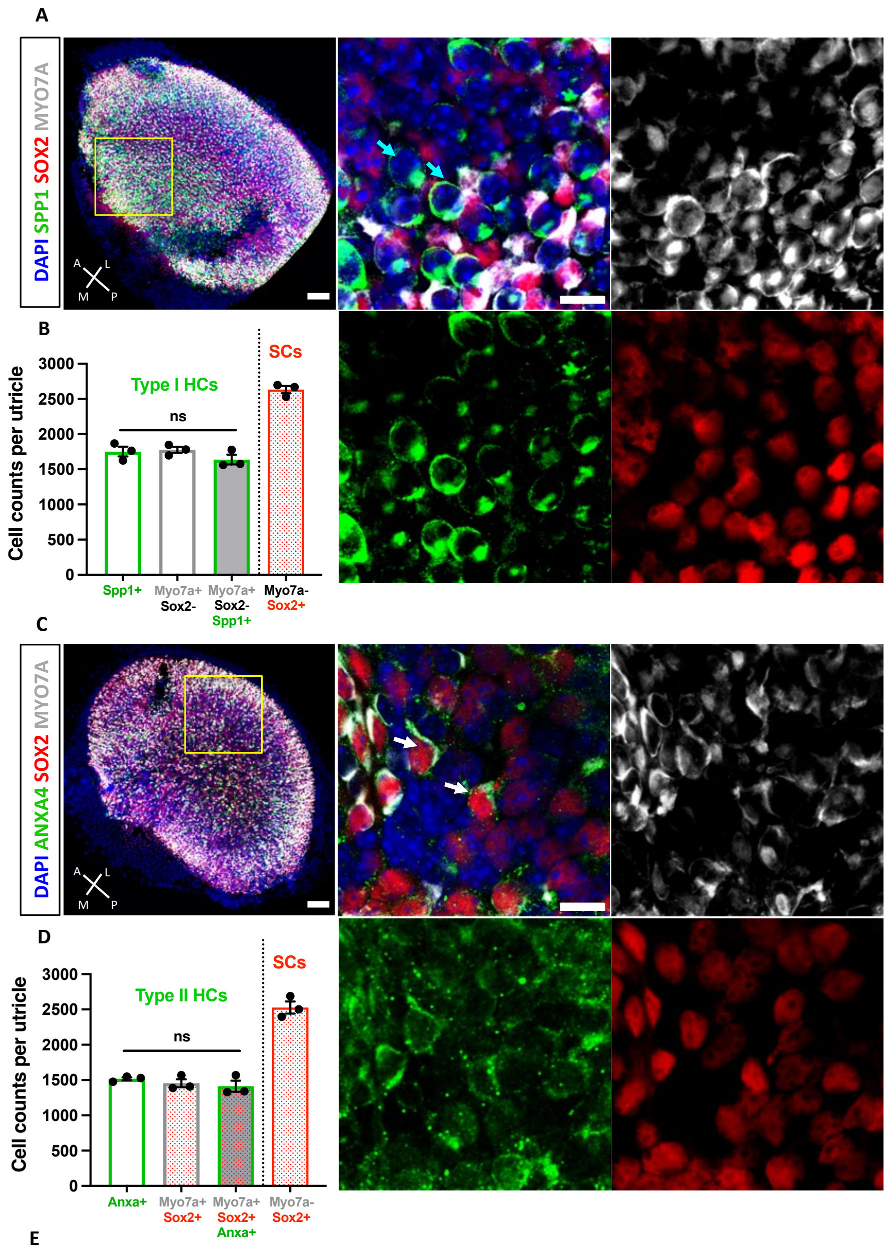

E

Cell counts (Mean ± SEM)	n	Type I HCs	Type II HCs	SCs
Lahlou et al,. 2024	3,3	1775 ± 42	1455 ± 57	2580 ± 50
Desai et al,. 2004	3	1789 ± 43	1457 ± 62	2754 ± 50

Supplemental Figure 3. SPP1 and ANNEXIN A4 label type I and type II HCs, respectively. Related to Figure 2. Utricles were harvested from 5-week-old WT mice and immunolabeled for SOX2 (red), MYO7A (gray), in combination with Spp1 or Annexin A4. Cell nuclei were stained with DAPI (blue). (**A**) Immunolabelling of type I HC marker SPP1, cyan arrows indicate type I HCs, negative for SOX2 (red), co-labeled with SPP1 (green) and MYO7A (gray). (**B**) Type I HC and SC counts. (**C**) Immunolabelling of type II HC marker ANXA4, white arrows indicate type II HCs co-labeled with ANXA4 (green), SOX2 (red) and MYO7A (gray). (**D**) Type II HC and SC counts. (**E**) Comparative table of type I, type II and SC counts to Desai et *al*, 2004. All data represent the mean ± SEM. Non-significant (ns) calculated by 1-way ANOVA with Tukey's multiple comparison test (**B** and **D**). Scale bars = 50 - 10 µm.

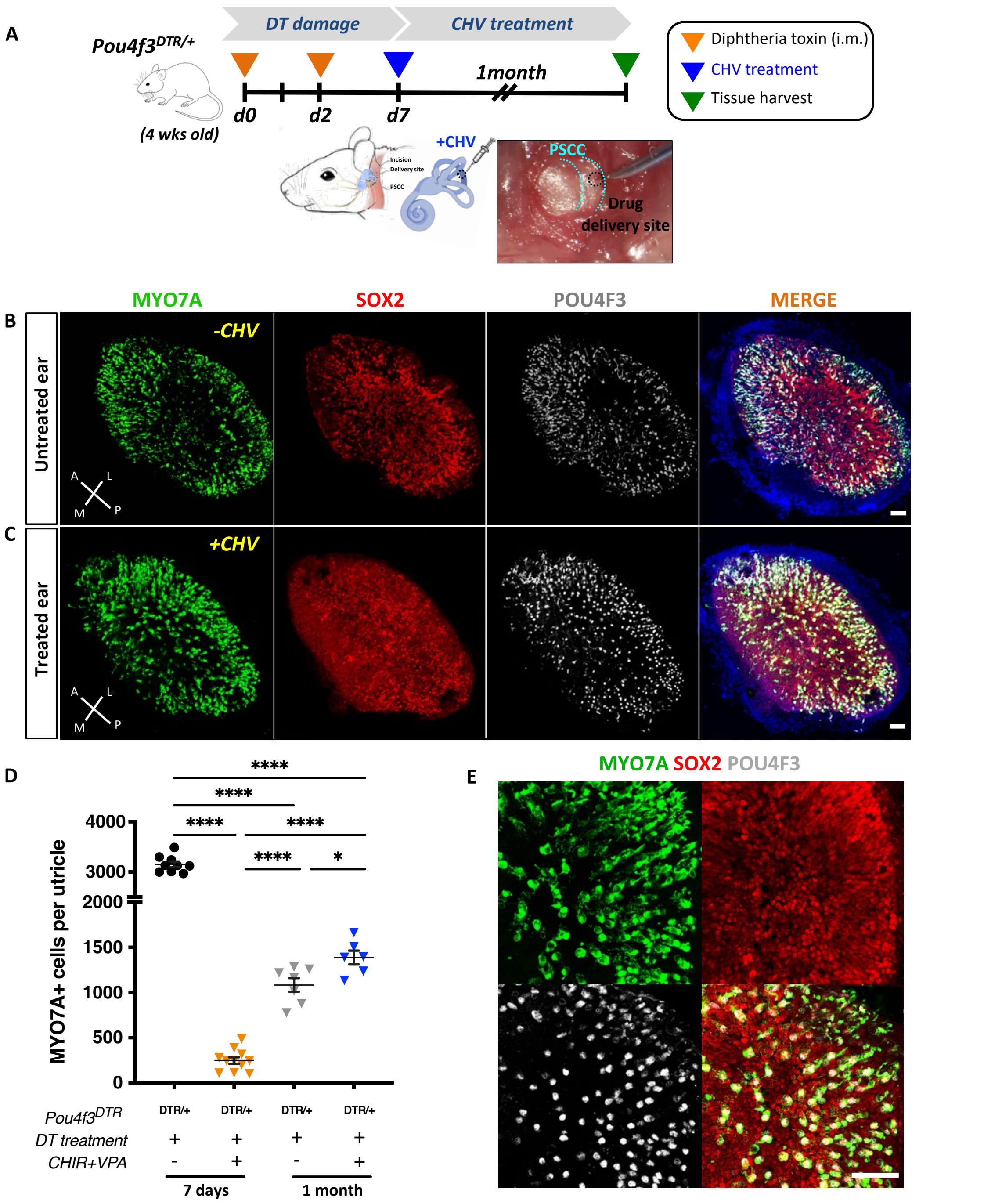

Supplemental Figure 4. HC regeneration 1 month after in vivo CHV treatment. Related to Figure 4. (A) Schematic of the in vivo approach for drug delivery in DT-ablated *Pou4f3*$^{DTR/+}$ mice. CHIR and VPA were injected in the ear via the semicircular canal 7 days after DT treatment; the contralateral ear was used as a control for spontaneous regeneration. Utricles were labelled with MYO7A (green), SOX2 (red) and POU4F3 (gray); cell nuclei were stained with DAPI (blue). (**B**) Immunolabelled utricles from the drug treated ear (+CHV). (**C**) Immunolabelled utricle from the untreated ear (-CHV). **(D)** Quantification of MYO7A immunopositive cells in WT, DT-ablated *Pou4f3*$^{DTR/+}$ without CHIR and VPA treatment (-CHV), and DT-ablated *Pou4f3*$^{DTR/+}$ utricles with drug treatment (+CHV). (**E**) High-magnification images from the CHV treated ear. All data represent the mean ± SEM. ****p < 0.0001, *p < 0.05 by 2-tailed Student's t tests and 1-way ANOVA with Tukey's multiple comparison test **(D)**. Scale bars = 50 - 100 µm.

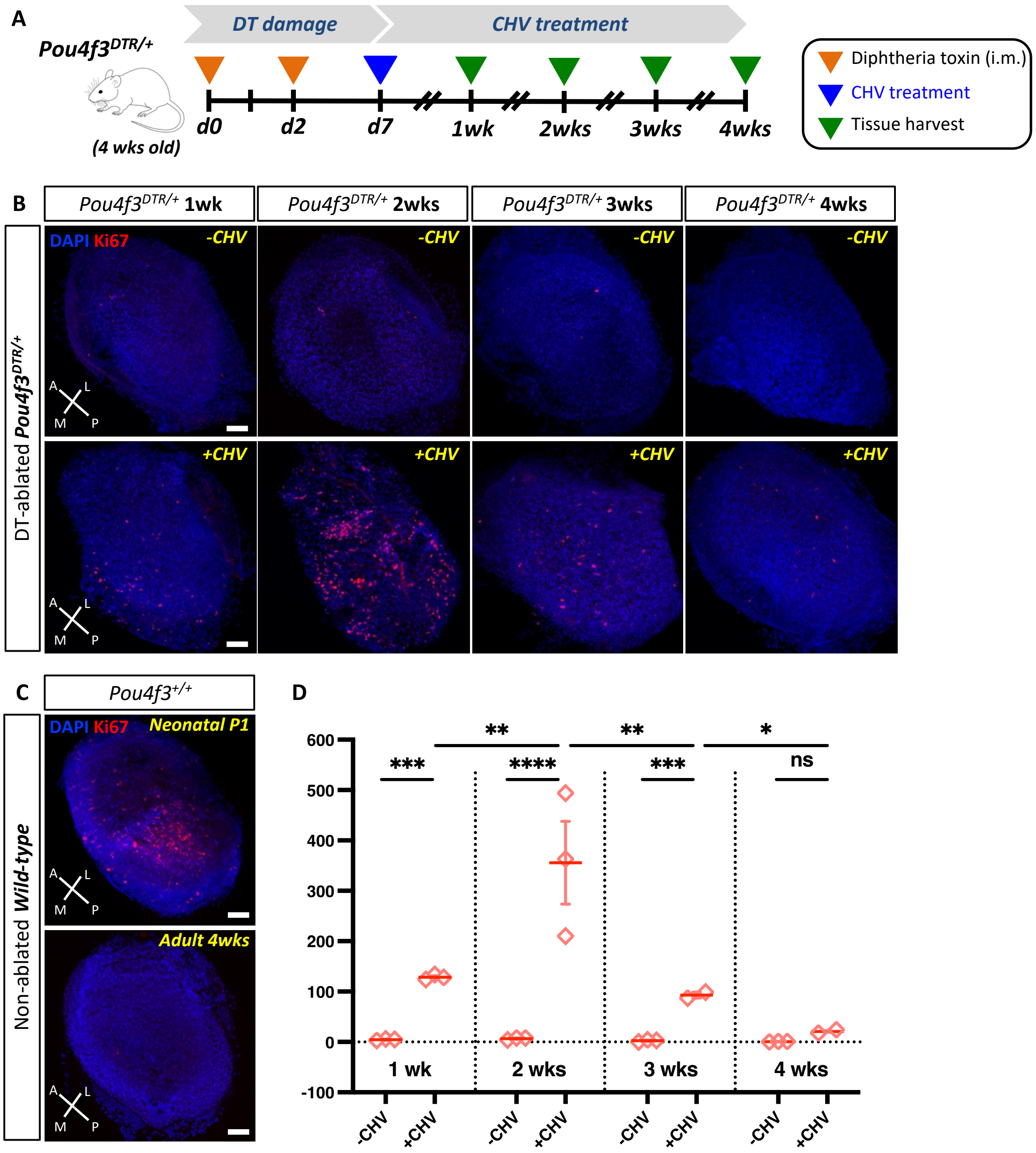

Supplemental Figure 5. In vivo CHV treatment stimulates proliferation in DT-ablated *Pou4f3*$^{DTR/+}$ utricles. (A) Schematic of the in vivo approach for drug delivery in DT-ablated *Pou4f3*$^{DTR/+}$ mice. CHIR and VPA were injected via the posterior semicircular canal (PSCC) 7 days after DT ablation; the contralateral ear was used as a control for spontaneous regeneration. (**B**) Ki67 (red) immunolabeling of adult *Pou4f3*$^{DTR/+}$ DT-ablated utricles from the untreated (-CHV) and treated (+CHV) ears at 1, 2, 3 and 4 weeks after treatment. (**C**) Ki67 (red) immunolabeling of undamaged neonatal (P1) and adult (4 wks) utricles. (**D**) Quantification of Ki67 immunolabeled cells from the untreated (-CHV) and treated (+CHV) ears at 1, 2, 3 and 4 weeks after treatment. All data represent the mean ± SEM. ****$p < 0.0001$, ***$p < 0.001$, *$p < 0.05$ by 2-tailed Student's t tests and 1-way ANOVA with Tukey's multiple comparison test **(D)**. Scale bar = 50 μm

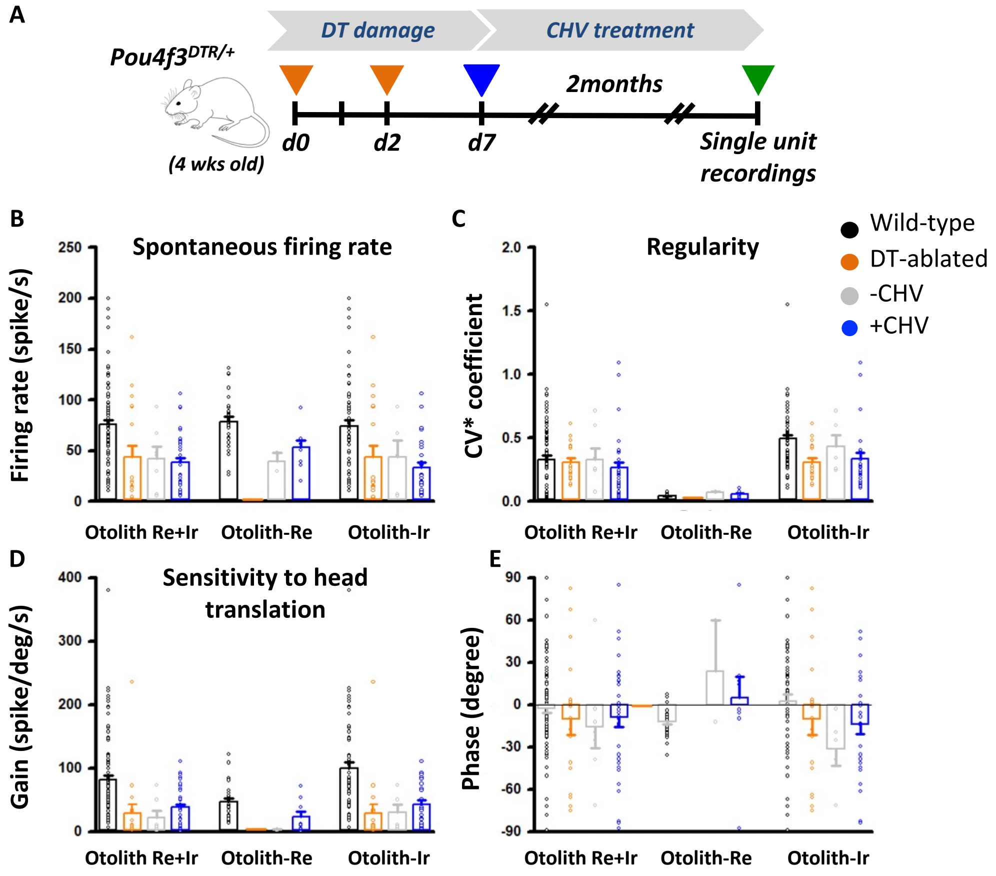

Supplemental Figure 6. Otolith afferent responses. Related to Figure 6. (**A**) Schematic of in vivo drug treatment of DT-ablated *Pou4f3*$^{DTR/+}$ mice. CHIR and VPA were injected via the semicircular canal at 7 days after DT treatment; the contralateral ear was used as a control for spontaneous regeneration. Mice were examined 2 months after drug treatment. (**B**) Averaged spontaneous firing rates. (**C**) Otolith afferent regularity. Normalized coefficient of variation (CV*) of inter-spike intervals. (**D-E**) Otolith afferents sensitivity to head translation gains (**D**) and phases (**E**).

A

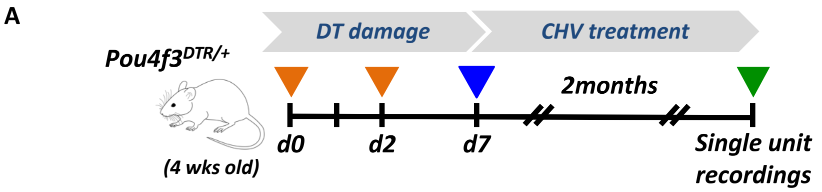

B

	Mice	All afferents		Canal afferents		% Canal afferents	
		Regular	Irregular	Regular	Irregular	Regular	Irregular
Wild-type group	n = 9	92	187	52	48	19%	17%
Ablated group	n = 6	15	111	1	0	1%	0%
-CHV group	n = 5	15	71	0	0	0%	0%
+CHV group	n = 5	21	91	1	7	1%	6%

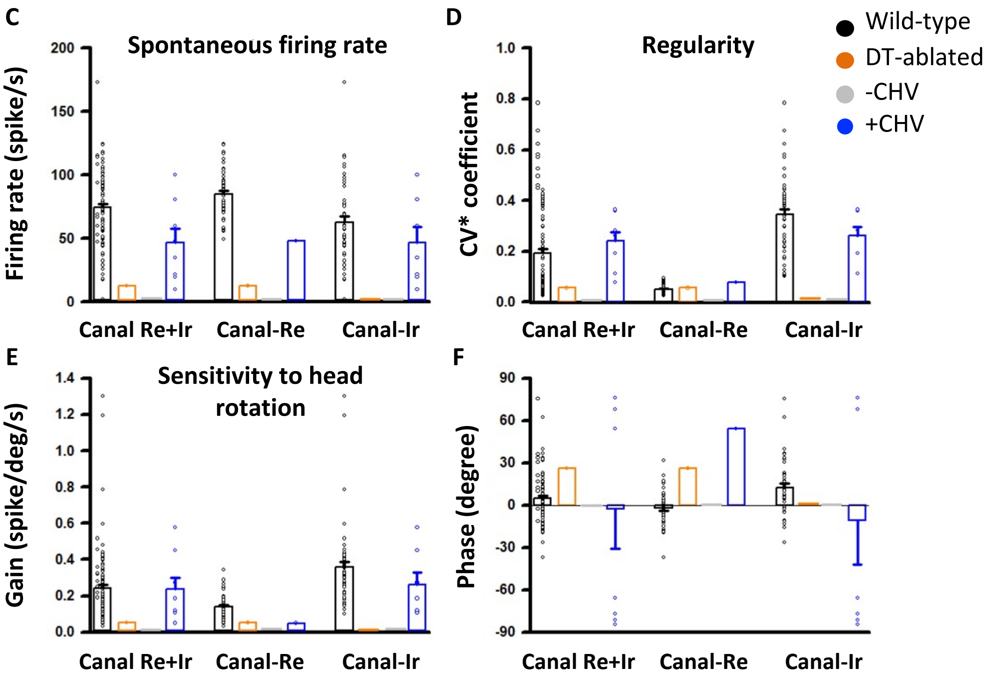

Supplemental Figure 7. Canal afferent responses. Related to Figure 6. (**A**) Schematic of in vivo drug treatment of DT-ablated *Pou4f3*$^{DTR/+}$ mice. CHIR and VPA were injected via the semicircular canal 7 days after DT treatment; the contralateral ear was used as a control for spontaneous regeneration. Mice were examined 2 months after drug treatment. (**B**) Table summarizing single unit recording of vestibular afferents. Canal afferent percentages are normalized by the total recorded afferents in each group. Percentages of WT and CHV treated canal afferents are boxed in red. (**C**) Average spontaneous firing rates. (**D**) Canal afferent regularity. Normalized coefficient of variation (CV*) of inter-spike intervals. (**E-F**) Canal afferents sensitivity to head rotation gains (**E**) and phases (**F**).

Supplemental Tables

Supplemental Table 1: Resources

REAGENT or RESOURCE	SOURCE	IDENTIFIER
**Antibodies**		
**Goat anti-ANNEXIN A4 (1:50)**	R&D system	*cat#AF4146*
**Rabbit anti-MYO7A (1:250)**	Proteus	*cat#25-6790*
**Goat anti-SPP1 (1:100)**	R&D System	*cat#AF8082*
**Mouse anti-POU4F3 (1:100)**	Abnova (interchim)	*cat#H-5459-M01 (DB9310)*
**Rat anti-SOX2 (1:100)**	Invitrogen	*cat#14-9811-82*
**Rabbit anti-TNC (1:500)**	Millipore	*cat#AB19013*
**Chemicals and drugs**		
**Tamoxifen**	Sigma Aldrich	T5648-1G
**Diphtheria Toxin (DT)**	Sigma Aldrich	D0564
**CHIR99021**	Cayman Chemical Company	13122
**VPA (Valproic Acid)**	Sigma Aldrich	P4543
**Polyethylene Glycol 400**	Covetrus	89510
**Buprenorphine Meloxicam**	Covetrus	055175
**Xylazine**	Covetrus	049755
**Ketamine**	Covetrus	061035
**DMSO**	Ketaved	071069
**DMEM/F12**	Sigma Aldrich	D8418
**B27**	Gibco	10565-018
**N2**	ThermoFisher	17504-044<br>17502-048
**Experimental Models: mouse strains**		
**C57Bl6**	The Jackson Laboratory	JAX #000664
**Pou4f3^{DTR}**	The Jackson Laboratory	JAX #028673
**Plp13^{CreRT}**	The Jackson Laboratory	JAX #005975
**mTmG**	The Jackson Laboratory	JAX #007676
**Genotyping Primers**		
Pou4f3^{DTR}	Common	AAGAAGCAGGTGGGGGAGAG
	WT Reverse	ATTGTTCTGGGCGACATGA
	Mutant Reverse	CAGAAAGAGCTTCAGCACCAC
Cre	Reverse	GGCCAGGCTGTTCTTCTTAG
	Forward	ATACCGGAGATCATGCAAGC
mTmG	Common	CTTCCCTCGTGATCTGCAAC
	WT Reverse	CAGGACAACGCCCACACA
	Mutant Reverse	GTTATGTAACGCGGAACTCCA
**Software and Algorithms**		
**MatLab (MathWorks, Natick, MA)**		
**SigmaPlot (Systat Software, San Jose, CA)**		
**Fiji-ImageJ**		
**GraphpadPrism**		

Supplemental Table 2: Single unit recording of vestibular afferents. Percentages are normalized to the total recorded afferents in each group. Comparison between untreated (-CHV, gray) and drug-treated (+CHV, blue) groups are highlighted in yellow.

	Mice	Total Afferents	Canal afferents		Otolith afferents		Unknown afferents	
**Wild-type group**	**n = 9**	**279**	**100**	**36%**	**87**	**31%**	**92**	**33%**
**Ablated group**	**n = 6**	**140**	**1**	**1%**	**18**	**13%**	**121**	**86%**
**-CHV group**	n = 5	104	0	0%	7	7%	97	93%
**+CHV group**	**n = 5**	**125**	**8**	**7%**	**36**	**29%**	**81**	**64%**
